# Skeletal muscle stem cells in comfort and stress

**DOI:** 10.1038/s41536-018-0062-3

**Published:** 2018-12-21

**Authors:** Brendan Evano, Shahragim Tajbakhsh

**Affiliations:** 1 Stem Cells and Development, Department of Developmental & Stem Cell Biology, Institut Pasteur, 75015 Paris, France; 2 CNRS UMR 3738, Institut Pasteur, 75015 Paris, France

## Abstract

Investigations on developmental and regenerative myogenesis have led to major advances in decrypting stem cell properties and potential, as well as their interactions within the evolving niche. As a consequence, regenerative myogenesis has provided a forum to investigate intrinsic regulators of stem cell properties as well as extrinsic factors, including stromal cells, during normal growth and following injury and disease. Here we review some of the latest advances in the field that have exposed fundamental processes including regulation of stress following trauma and ageing, senescence, DNA damage control and modes of symmetric and asymmetric cell divisions. Recent studies have begun to explore the nature of the niche that is distinct in different muscle groups, and that is altered from prenatal to postnatal stages, and during ageing. We also discuss heterogeneities among muscle stem cells and how distinct properties within the quiescent and proliferating cell states might impact on homoeostasis and regeneration. Interestingly, cellular quiescence, which was thought to be a passive cell state, is regulated by multiple mechanisms, many of which are deregulated in various contexts including ageing. These and other factors including metabolic activity and genetic background can impact on the efficiency of muscle regeneration.

## Introduction

Regenerative myogenesis has emerged as arguably one of the most powerful paradigms to investigate a variety of processes involving stem cells and tissuegenesis. Adult skeletal muscle satellite (stem) cells emerge from a proliferative population of myogenic cells that reversibly exit the cell cycle asynchronously during perinatal growth.^1,2^ They are located between muscle fibres and the basement membrane ensheathing it and since their initial identification in the frog,^3^ genetic and cell lineage strategies led to detailed analysis of their properties. Notably, critical regulators of quiescence, commitment and self-renewal have been identified, while exposing underlying heterogeneities in myogenic cell states.^1,4^

In vertebrates, genetic and embryological studies have shown that the bHLH myogenic regulatory factors (MRFs) *Myf5*, *Mrf4*, *Myod* and *Myogenin* have crucial roles in governing striated muscle cell fate and differentiation. Mice triple mutant for *Myf5*, *Myod* and *Mrf4* lack skeletal muscles and their progenitors pointing to these genes as critical determination factors, whereas *Myogenin* and *Mrf4* act during differentiation.^4–7^ In the adult, *Myf5* is expressed in quiescent and activated satellite cells,^8^ whereas MYOD protein expression is a hallmark of an activated satellite cell.^4–6^ Upstream transcription factors include *Pax3*, *Tbx1*, *Six1/4* and *Pitx2* and they act in different locations in the embryo to establish the founder muscle stem cell population.^9^ The properties of the paraxial mesoderm from which head and body muscles arise are also different. All body muscles and some located in the head arise from transient structures called somites, and these are under the regulation of the paired/homeobox transcription factors *Pax3* in the embryo, and later, *Pax7*.^10,11^ In contrast, head muscles are derived from cranial paraxial mesoderm and are *Pax3*-independent, but regulated in the embryo by *Tbx1* and *Pitx2* among other genes.^9,12^ From mid-embryonic stages, virtually all stem/progenitor cells throughout the body are marked by *Pax7* expression. These cell-intrinsic differences observed during embryogenesis occur in the context of a heterogeneous extrinsic milieu. Indeed, an important consideration is the role of stromal cells that constitute the stem cell niche, and that also arise from distinct embryological origins in the head and the body. Their impact on muscle stem/progenitor cell fates remains largely unexplored. Some of these issues will be discussed in this review.

## The interplay between satellite and stromal cells in skeletal muscle

**Fig. 1:**
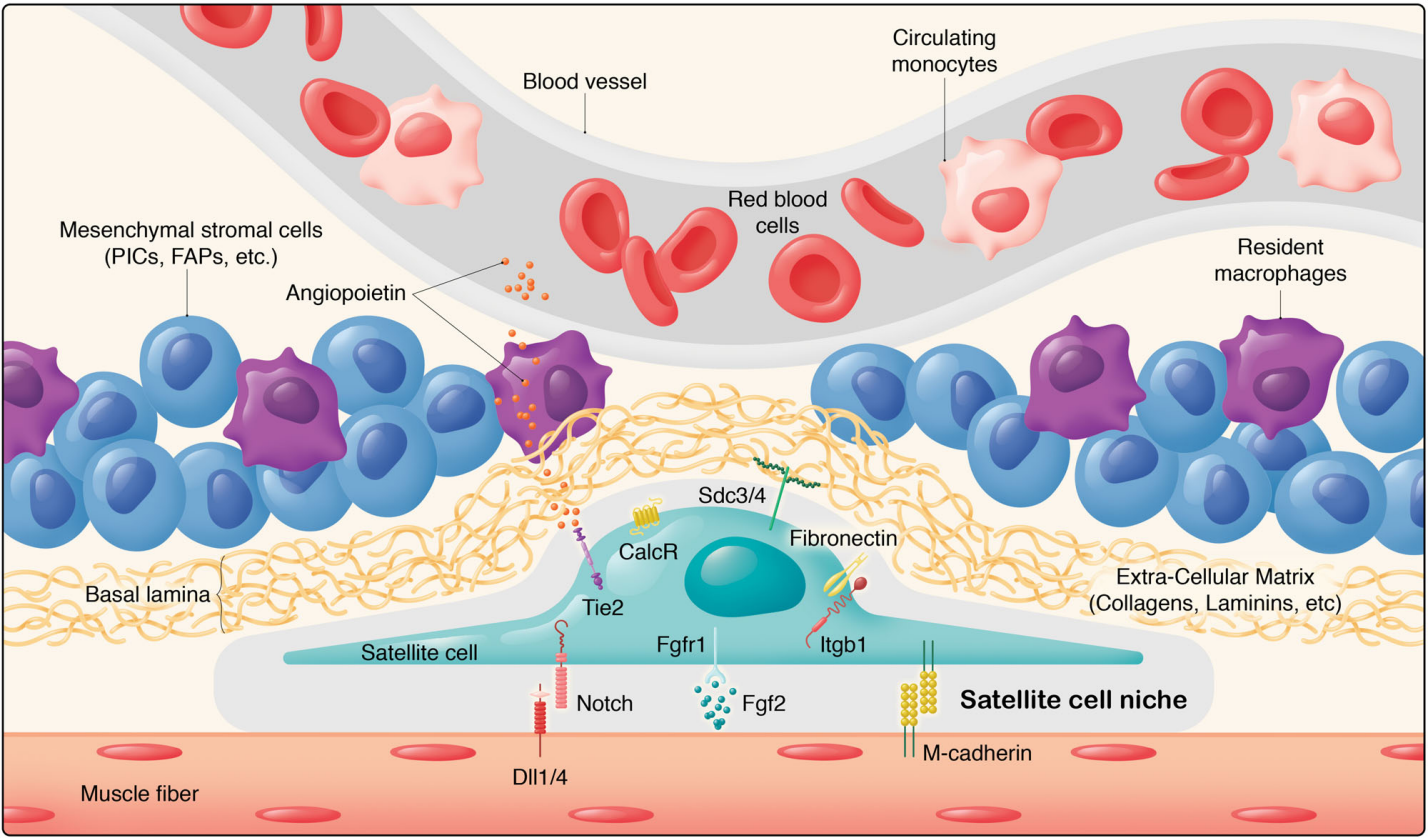
The satellite cell and stromal cell niche. Satellite cells states are regulated through their interactions with their microenvironment. While direct interactions (M-cadherin, Notch pathway)^38,46^ and communication (FGF2-FGFR1 pathway)^48^ between muscle fibres and satellite cells have been identified, muscle stem cells also interact with a variety of components of the extracellular matrix (e.g. Collagens VI and V, Laminin, Fibronectin, SDC3/4)^45,79^ and diffusable cytokines and growth factors (e.g. Angiopoietin-Tie2 receptor)^37^. In addition to satellite cells, several cell types contribute to muscle growth, homoeostasis and regeneration, including pericytes, mesenchymal stromal cells (e.g. Pw1+ Interstitial Cells, FibroAdipogenic Progenitors, Twist2+ cells)^16,19,21^, immune cells (e.g. resident or infiltrating macrophages)^156^ as well as connective tissue cells. These interactions are remodelled during ageing, notably with increased FGF2 production from muscle fibres and decreased expression of FGFR1 in satellite cells, driving satellite cells to break quiescence^39^, and decreased levels of fibronectin^45^, which weakens satellite cell adhesion capacity and increases their susceptibility to apoptosis by anoikis

Although fewer studies have focused on the role of interstitial cells (Fig. 1), their critical roles in homoeostasis and regeneration has been highlighted in several reports. For example, fibroadipogenic progenitors (FAPs) promote myoblast differentiation and participate in fibrosis following muscle damage.^13,14^ Another cell type, called PICs (Pw1 + interstitial cell) was also identified as residing outside the basement membrane of the muscle fibre. *Pw1* is an imprinted gene that is involved in stress regulation.^15,16^ The transplantation of PICs into injured muscle results in their contribution to regenerating fibres.^16^ Mesoangioblasts that are associated with blood vessels were also reported to contribute to skeletal muscles.^17^ Interestingly, mesoangioblasts isolated from mouse, dog and human express high levels of *Pw1*, where this gene was shown to confer the myogenic potential of mesoangioblasts, and their ability to cross the vessel wall.^18^ Recently, an interstitial cell type that is marked by the basic-Helix-loop-Helix transcription factor *Twist2* (*Dermo1*), was reported to be *Pax7*-negative during homoeostasis and following muscle injury.^19^ Intriguingly, these cells contribute specifically to type IIb/x myofibres during adulthood and muscle regeneration, and their genetic ablation causes wasting of type IIb (fast glycolytic) myofibres.^19^ How these different cell types are related to so-called “mesenchymal stem cells” remains obscure. FAPs can be isolated by cytometry using PDGFRα/Sca1^20^ whereas PICs (PW1+/PDGFRα− fraction with myogenic capacity) were reported to be a sub-population of interstitial cells.^21^ As the field tries to resolve these different cell types further, it is interesting to note that a detailed study of mesenchymal “stem” cells pointed to a substantial heterogeneity in this population depending on their tissue of origin,^22^ therefore a concerted effort is clearly needed to further characterise stromal cells in different tissues and address the potentially misleading designation of stromal cells generically as “mesenchymal stem cells”. Given these findings, and the potential misappropriation of cell populations, single cell mass spectrometry and single cell RNAseq of the entire muscle resident cell population was done to resolve some of these discrepancies.^23^ Of note a total of 10 different cell types were identified, including known populations (satellite cells, FAPs, endothelial cells, etc) and previously uncharacterised resident tenocyte-like cells and smooth muscle/mesenchymal cells with myogenic potential were identified.^23^

More detailed studies on FAPs have shown that TNFα-mediated apoptosis of this population is critical for normal regeneration. During chronic injury, as with dystrophic *mdx* mice, continued expression of transforming growth factor β1 (TGF β1) results in persistence of FAPs and fibrosis. Pharmacological inhibition with the tyrosine kinase inhibitor Nilotinib, which has potent antifibrotic activity, blocks TGF β1 activity and results in reduced fibrosis.^20^ However, Nilotinib treatment also blocks expansion of FAPs and compromises regeneration through a non-cell-autonomous anti-proliferative effect on satellite cells.^24^ These studies and others cited below highlight the dynamic nature of regeneration, and the importance of determining when to intervene for a desired outcome.

Stromal cells that have myogenic capacity have been considerably less well characterised compared to satellite cells, and their contributions to muscle or self-renewal into a satellite cell position are limited, or not demonstrated, compared to bona fide satellite cells. Significantly, elimination of satellite cells by selective diphtheria toxin ablation results in failed regeneration,^25–27^ indicating that in the short term, non-satellite cells do not contribute to muscle regeneration. Similar ablation studies should be extended to all interstitial cell types.

**Fig. 2:**
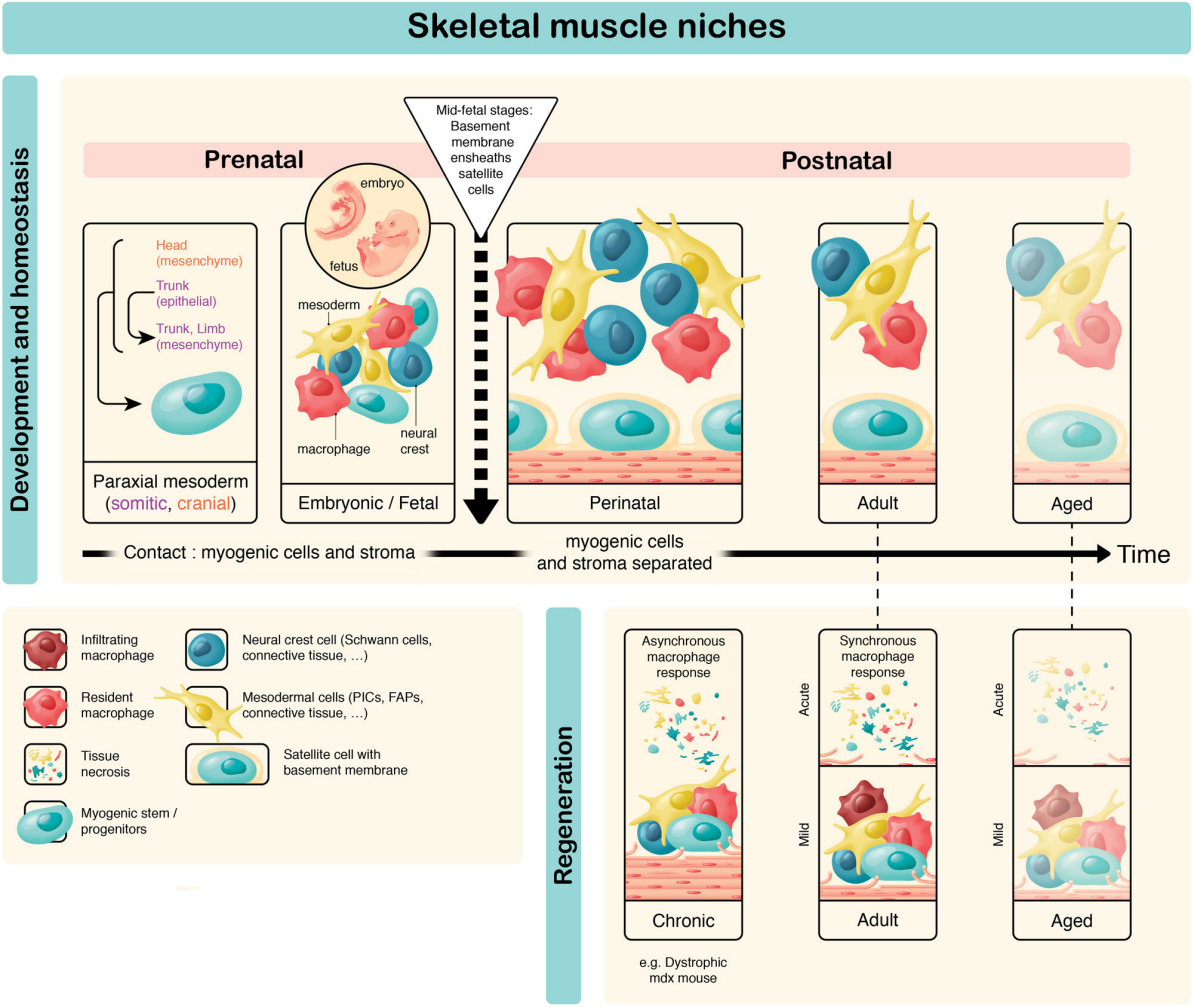
Developmental, adult, ageing and diseased skeletal muscle niches. During development and regeneration, stromal and myogenics cells can be in direct contact, and myogenic cells can be exposed to stromal-derived extracellular matrix proteins, whereas in the late foetal to postnatal stages, muscles stem cells are separated from stromal cells by a basement membrane (from mid-foetal stages)^1,28^. Stromal cells have distinct embryonic origins depending on their anatomical location—those in the head are of neural crest and mesoderm origin, whereas those in the limbs are mostly of mesodermal origin^4^. In addition to this spatial character, niche cells evolve during development and postnatal life thereby introducing a temporal dimension to the regulation of muscle stem cells as they give rise to quiescent satellite cells and age. The postnatal niche is disrupted following chronic (e.g. myopathies) and acute (chemical, physical) injury. In the former, there is an asynchronous response of infiltrate and in the latter, more phasic appearance and disappearance of neutrophils and macrophages is noted

It is interesting to note that satellite cells have significantly distinct genetic requirements in different anatomical locations as indicated above (e.g. *Tbx1/Pitx2* in head; *Pax3* in body). It is therefore likely that stromal cells, which can be of mesodermal or neural crest origin, might impact differentially on the fate of myogenic cells in relation to their anatomical location.^4^ Importantly, emerging satellite cells continue to proliferate until about 2 weeks postnatally, yet they are ensheathed under a basement membrane from mid-late foetal stages.^1,28^ Therefore, contact with extracellular matrix proteins in the basal lamina of the basement membrane is not sufficient to trigger cell cycle exit. In addition, enseathment under the basal lamina results in pre-quiescent and post-quiescent satellite cells being physically separated from stromal cells and in contact only with the myofibre, until its disruption following injury (Fig. 2). How sporadic interactions with stromal cells prior to this confinement affect the fate of myogenic cells is an open question. Given that the transcriptome profiles of *Pax7*+ myogenic cells change significantly during prenatal and postnatal development,^29–31^ stem cell and niche cell interactions need to be explored further in different contexts when direct satellite and niche cell contacts can occur (Fig. 2).

## Identification of the quiescent state

Non-cycling cells have been characterised in different states: quiescent, terminally differentiated, apoptotic/necrotic, and senescent. In contrast to apoptosis, differentiation and senescence—cellular quiescence is a reversible cell state representing a non-cycling cell. In multicellular organisms, postnatal tissue homoeostasis and regeneration upon trauma are mediated by adult tissue specific stem cells that are generally quiescent. Following injury, and in response to diffusible and mechanical cues, adult satellite cells activate to generate transit amplifying myoblasts that will in turn fuse to form multinucleated myofibres. A subset of the transit amplifying precursors renews the stem cell pool by returning to the quiescent state. In vitro models can recapitulate this process to some extent where a pool of ‘reserve’ cells is associated with differentiated myotubes.^32^ Efforts have also been made to artificially recapitulate cellular quiescence or the niche environment ex vivo.^33–35^

In the yeast *Saccharomyces cerevisiae*, quiescence is induced in response to nutrient limitation.^36^ In multicellular organisms, cellular quiescence might also act as a protective mechanism, against accumulation of mutations due to proliferation, but also against various environmental stresses. Deregulation of quiescence can lead to precocious differentiation, senescence, or apoptosis, and it is associated with impaired tissue regeneration.^37–51^ In the context of therapies, a promising strategy is the transplantation of tissue stem cells that have been corrected in vitro. To date, a major challenge has been overcoming the loss of stemness properties following ex vivo expansion,^52,53^ and diverse strategies,^54–56^ including isolation of transplantable myogenic cells from teratomas,^57^ show some promising results. Furthermore, retaining quiescence properties might be a means to survive the transplantation process,^58,59^ thereby underscoring the importance of characterising this cell state.

**Fig. 3:**
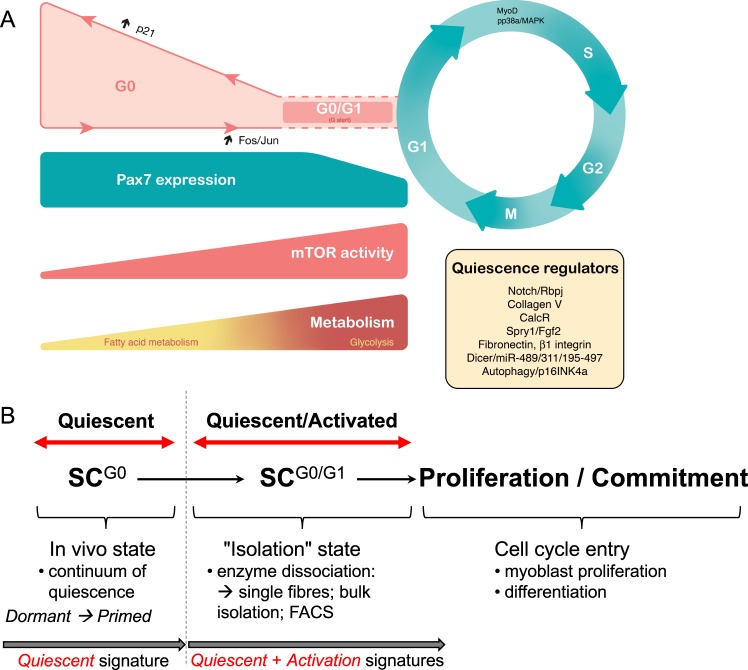
Quiescence to proliferation transition in satellite cells. **a** During homoeostasis, adult satellite cells are maintained in a reversible non-proliferating quiescent G0 state by regulators including Calcitonin receptor, Collagen V, Notch pathway, FGF signalling and effectors of the RNAi machinery^39,40,46,51,79^. However, specific quiescence markers are still lacking. Satellite cells within a healthy tissue respond to a distant injury by transiting from deep quiescence to a quiescent G0/G1 or ‘G(alert)’ state^85^, with increased proliferative capacity and regenerative potential. This transition is under the control of mammalian target of rapamycin (mTOR) signalling, which in turns controls mitochondrial metabolism (see text). Following an acute tissue injury or chronic mild degeneration of muscle fibres, satellite cells exit from their quiescent state and proliferate. This transition is accompanied by a metabolic shift from fatty acid oxidation to glycolysis.^147^ Some cells irreversibly exit the cell cycle to differentiate into mononuclear myocytes that eventually fuse to regenerate muscle fibres, while others self-renew and return to quiescence. Entry into quiescence is poorly characterised, and an activation marker is MYOD. **b** Isolation of adult quiescent satellite cells involves repeated mechanical and enzymatic dissociation of the tissue. These procedures invariably lead to satellite cell activation, as shown by the rapid upregulation of FOS and JUN, and phosphorylation of p38^65^

Cellular quiescence, or G0, is defined as a transient and reversible cell cycle arrest during the G1 phase (Fig. 3). It is characterised by a decrease in cell size, increased nucleocytoplasmic ratio, 2N genome content, low RNA and protein synthesis, altered metabolism and gene expression profile.^60^ Quiescence had been considered for some time to be a default state corresponding to the absence of proliferation and differentiation. It is now clear that cellular quiescence is an actively maintained state^61^ with new functional markers continually being identified.

Considerable effort has been put into identifying quiescence-specific markers, notably for satellite cells^31,41,45,62−67^ (Fig. 3a). Of those, *Calcr* (Calcitonin receptor) and *Odz4* are of interest as they encode membrane-associated proteins that could potentially be used to isolate quiescent muscle stem cells by fluorescence activated cell sorting (FACS).^68,69^ Interestingly, *Odz4* is re-expressed earlier than *Calcr* in satellite cells during regeneration,^69^ and proliferating neonatal satellite cells express *Odz4*, but not *Calcr*, indicating that some quiescence genes are differentially expressed during homoeostasis, muscle regeneration and postnatal growth.

Until recently, studies focused on identifying quiescence-specific transcripts in adult muscle stem cells have required purification procedures based on mechanical and enzymatic dissociation followed by satellite cell isolation by FACS. These involve dissociating satellite cells from their microenvironment, which invariably leads to their activation (Fig. 3b). In addition, it is now clear that satellite cell activation occurs more rapidly than previously thought; for example phosphorylated p38 appears within 30 min after isolating satellite cells on myofibres.^70^ Hence, some highly dynamic quiescence-specific or activation-specific genes, non-coding RNAs, and epigenetic states might have been missed in previous studies^31,65,67^ (Fig. 3b). Accordingly, it has been known for decades that the Fos/Jun stress response pathway activates within minutes during the G0 to G1 transition or in response to stress^31,71^ (Fig. 3b). Although some markers allow the identification of deeply quiescent satellite cells and of actively proliferating myoblasts, in vivo dynamic markers to follow early activation or re-entry into quiescence during regeneration are largely lacking.

## Quiescent muscle stem cells

### Molecular regulation of quiescence

Notch signalling has a critical role in maintaining quiescence of satellite cells, and the expression of canonical Notch targets (e.g. *Hes*, *Hey*) is markedly reduced before cells enter the cell cycle, thereby allowing MYOD protein to accumulate.^20,28^ Later during regeneration, Notch signalling increases, both in differentiating and in self-renewing myoblasts.^72^

During homoeostasis, quiescent satellite cells display high levels of Notch pathway activity, relayed by its intracellular effector *Rbpj*. Satellite cell-specific deletion of *Rbpj* during homoeostasis results in loss of satellite cells, their differentiation, and fusion to existing myofibers.^38,46^ Intriguingly, in this case the majority of satellite cells boycott S-phase and do not enter the cell cycle (absence of BrdU uptake). Deletion of *Rbpj* also leads to an impaired regeneration capacity following injury.^46^ Nonetheless, *Rbpj* null satellite cells can undergo a normal activation and transit amplification after muscle injury, indicating different contextual roles for Notch signalling in maintenance of quiescence and initial cell cycle progression through G1/S in muscle homoeostasis or regeneration.^72^ Another role for Notch was proposed, this time through regulation of E3 ubiquitin ligase *Mib1* (Mind bomb 1) which triggers endocytosis of ligands that interact with Notch receptor.^73^ Remarkably, sex hormones were reported to act during puberty, and after regeneration, to induce *Mib1* in myofibres, thereby resulting in increased Notch activation in satellite cells, and their entry into quiescence. This model might appear counterintuitive, as it suggests that satellite cell quiescence in different muscle masses is under systemic regulation. Moreover, Notch activation inhibits expression of *Myod* and sustains the expression of *Pax7* while allowing the proper homing of satellite cells to their niche, thereby participating in the maintenance of the non-committed state of satellite cells.^46,74,75^

As mentioned above, *Odz4* is specifically expressed in neonatal and adult quiescent satellite cells. The ODZ proteins are type II transmembrane proteins, for which protein cleavage sites have been demonstrated in the extracellular and transmembrane domains, and cleaved intracellular ODZ2 acts as a transcription factor.^76^ Although the mode of action of *Odz4* specifically in satellite cells remains to be determined, *Odz4* germline null mice display a reduced body weight and size, decreased muscle mass and satellite cell pool size, both during homeostasis and after injury-mediated regeneration. Satellite cells isolated from these animals show prolonged *in vitro* proliferation and enhanced differentiation.^77^

In addition, a satellite cell-specific deletion of *Calcr* sensitises satellite cells to apoptosis, which eventually decreases the size of the stem cell pool and impairs regeneration efficiency upon injury.^51^ *Calcr* encodes a G-protein coupled receptor (GPCR), known to regulate calcemia, and CALCR and its ligand calcitonin inhibit bone resorption in osteoclasts.^78^ In muscle, *Calcr*^−^^/−^ satellite cells express higher levels of the cell cycle marker KI67 and other cell-cycle-related genes (*Ccna2*, *Ccnd1*), without any increase in expression of myogenic genes such as *Myod*, and without impacting on myogenic differentiation.^51^ Interestingly, *Calcr* was reported to regulate satellite cell quiescence through cAMP-PKA signalling and thereby position satellite cells within their niche through cAMP-Epac signalling.^51^ Intriguingly, the ligand for CALCR, calcitonin, is produced by the thyroid, therefore, as for the regulation of *Mib1* indicated above, this suggests that maintenance of satellite cell quiescence is also under systemic control. However recent evidence from our laboratory suggests that this may not be the case following identification of a novel *Notch*/*ColV*/*Calcr* axis where Collagen V, expressed by satellite cells, can act as a surrogate ligand for CALCR to maintain the quiescent state cell-autonomously.^79^

Interestingly, intron 4 of *Calcr* contains the microRNA miR489, which is necessary for their maintenance of the quiescent state by post-transcriptionally repressing the oncogene *Dek*.^40^ In addition, miR-195/497 was shown to induce cell cycle arrest^80^ suggesting that other miRs could have critical roles in maintenance of quiescence. Indeed, satellite cell loss of *Dicer*, which is a key mediator of miRNA processing, resulted in depletion of this pool, upregulation of KI67, and failure to regenerate following injury.^40^

Other signalling pathways have been explored in the context of satellite cell quiescence. For example, Angiopoietin 1 (ANG1) and its receptor TIE-2 regulate the quiescent state where TIE-2 is expressed by a subset of quiescent satellite cells and ANG1/TIE-2 signalling, through ERK1/2 pathway favours cell cycle exit.^37^ Furthermore, previous studies had suggested that c-MET and its ligand hepatocyte growth factor could regulate the quiescence to activation transition, however, conditional ablation of the *c-Met* receptor gene in satellite cells showed no requirement for satellite cell activation, myoblast proliferation, or myocyte differentiation, but a role in muscle fusion and regeneration.^51^ Oncostatin M, which belongs to the interleukin-6 family of cytokines, also regulates satellite cell quiescence as depletion of its receptor leads to loss of this stem cell population and impaired regeneration after injury.^81^

Although some transcripts of activated and differentiated cells are detected in quiescent satellite cells, their proteins are generally detected after activation. As indicated above, MYOD is one such example where appearance of the protein identifies, and can promote satellite cell activation. In this context, repression of translation can be critical for maintaining satellite cell quiescence. This is mediated by phosphorylation of translation initiation factor eIF2a, resulting in stabilisation of the quiescent state, as inhibition of eIF2a phosphorylation results in exit from quiescence, activation of the myogenic programme and compromised self-renewal.^82^

Intriguingly, a decrease in the satellite cell pool can be associated^40,46,51^ or not^48^ with impaired regeneration capacity upon injury. Indeed, a recent report indicated that the dynamics and extent of regeneration, both at the stem cell population and histological levels, vary depending on the method used to induce muscle injury^43^ (snake venom myotoxins, chemicals or physical procedures). Furthermore, the number of satellite cells can increase several fold with some injury procedures raising further questions on how stem cell numbers are regulated locally, and globally. Other factors, such as the myofibre, can impact on satellite cell numbers. For example, transgenic mice overexpressing *Tead1* (TEA domain transcription factor binds to Hippo signalling effectors YAP/TAZ) in myofibres have 6X more quiescent satellite cells.^83^ Although this transgenic mouse ameliorated muscle regeneration in a dystrophic *mdx* background, it is not clear if satellite cell numbers have a role. Taken together, these reports raise the question—how many stem cells are required to assure proper muscle function during regeneration and ageing?

### Distinct states of quiescence

Until recently, cellular quiescence was considered to be a passive and homogenous cellular state with reduced metabolic activity. Of note, recent reports indicate that quiescent satellite cells can exist in different states, more or less primed for commitment,^84^ or poised for activation.^85^ Satellite cells expressing high levels of *Pax7* are less primed for commitment, display a lower metabolic activity and a delayed first mitosis upon activation in vivo and in vitro compared to cells expressing lower levels of *Pax7*. PAX7^Hi^ cells were therefore proposed to be in a deeper quiescent, or “dormant” cell state, compared to the remaining population that is more primed for cell-cycle entry.^84^ Hence, the quiescent state exists as a continuum from PAX7^Hi^ to PAX7^Low^ cells.

During extreme stress, such as death, where cells are exposed to tissue necrosis, acidosis and lack of oxygen, the majority of satellite cells adopt a dormant cell state^86^ similar to that noted in a sub-population (PAX7^Hi^) in living mice.^84^ Therefore, satellite cells can modulate their physiological and metabolic status to adapt to changing microenvironments. It is likely that satellite cells that are primed would be first-responders to mild-injury, and that dormant satellite cells would be mobilised under severe trauma, however, this remains to be proven formally. By analogy, yeast that are subjected to nutrient deprivation alter their metabolic and transcriptome profiles significantly depending on the type of starvation regime employed.^36^ Therefore, one possibility is that dormancy vs. primed quiescence are determined by anatomical position. It is interesting to note that about 85% of satellite cells are located in close proximity to a blood vessel, whereas the remainder are distal, and likely in a more nutrient-deprived hypoxic niche,^87^ raising the possibility that these distally located cells might be in a dormant state.

Interestingly, quiescent satellite cells that are distant from a site of muscle injury (ex. contralateral muscle) can transit from G0 to the G0/G1 state (also called ‘G(alert)’), characterised by an increased cell size, mitochondrial activity, gene expression profile, improved differentiation and enhanced regeneration capacity^85^ (Fig. 2a). Although the BaCl_2_ that was used to promote muscle injury could have diffused to distal muscles, some of the observed phenotypes were confirmed with physical injury suggesting a systemic relay mechanism. This G0-G(alert) transition state is reversible and was shown to be dependent on the mTor signalling pathway.^85^ Taken together, these findings indicate that adult satellite cells exist in different quiescent states within which they can transit in response to environmental cues.

Following exit from quiescence, the fate a satellite cell can vary according to the context: apoptosis,^40,51^ direct differentiation^46^ or senescence.^41,49^ These cell states can be altered significantly during ageing, leading to a decline in satellite cell number and function^39,49,88,89^ as a result of defects in self-renewal, maintenance of quiescence, regenerative capacity or increased susceptibility to apoptosis and senescence.^39,41,45,49^

## Deregulation of satellite cell function: ageing and senescence

**Fig. 4:**
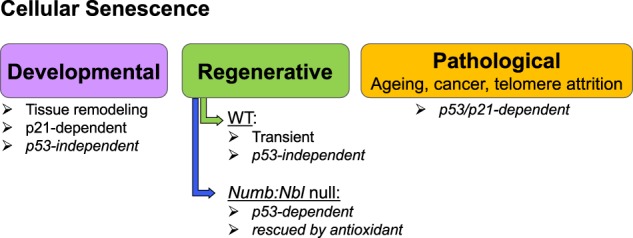
Cellular senescence in different contexts. Although senescence has been extensively reported in pathological contexts^90–92^, recent studies have reported that cellular senescence is associated with developmental and regenerative processes, suggesting a beneficial role^91,93–98^. Senescence observed during muscle regeneration was not altered on a *p53*-null background, however, *Numb:Numblike* mutants that exhibit a higher level, and persistent cell senescence during regeneration, are rescued on a *p53-null* background and with antioxidants suggesting that senescence can be modulated differentially in this mutant compared to wild-type mice^93^

Pathologies associated with ageing involve cell-autonomous as well as non-cell-autonomous mechanisms, including cellular senescence. Cellular senescence represents an irreversible cell cycle exit state that is often associated with pathologies where cancerous, DNA-damaged or ageing cells withdraw from the cell cycle and suppress apoptotic mechanisms while engaging tumour suppressors such as P53, P16/P19.^90–92^ An important signature of senescent cells that undergo a stress response such as oxidative, replicative and genotoxic stress, is the so-called “senescence-associated secretory phenotype” (SASP) where several growth factors, inflammatory molecules (chemokines, cytokines), proteases, and extracellular matrix components modify the fate of nearby cells. Currently, markers specific for senescent cells are lacking; commonly used markers include senescence-associated β-galactosidase (SA-β-Gal) activity in lysosomes, the level of lysosomal content, and elevated levels of P16.^90,91^ Although the pathological consequences of cellular senescence have been well documented where suppression of senescent cells can have dramatic consequences including increase of lifespan, its potential beneficial effects have only been reported recently for tissue remodelling during development and regeneration^91,93–98^ (Fig. 4). Suppression of senescence during prenatal stages does not promote overt phenotypes, however, removal of senescent endothelial and fibroblast cells was associated with less efficient wound healing of skin.^97^ These studies raise the notion of a beneficial role for cellular senescence, likely through the SASP (Fig. 4). Similarly, senescent cells were reported to have a role during limb regeneration in salamander following amputation.^98^ Furthermore, mutation of the endocytic adaptor proteins *Numb:Numblike* resulted in senescence of myogenic cells and compromised regeneration. This phenotype was rescued in a *p53*-null context, and by administration of an antioxidant.^93^ Notably, this *Numb*-mediated senescence was distinct from senescence observed in endothelial cells during early regeneration that was independent of *p53* and oxidative stress.^93^ It is interesting to note that cellular senescence also has a role in cell plasticity where the generation of teratomas following induction of pluripotency factors (*Oct4*, *Sox2*, *Klf4* and *Myc*) in vivo and ex vivo was modulated, at least in part, by senescent cells.^95^

Recent studies pointed to functional differences between aged and ‘geriatric’ mice where in the latter, the regenerative capacity of satellite cells decreases even further during extreme ageing, after 28 months of age in mice. Here, satellite cells lose their quiescent state to become pre-senescent, due to de-repression of the senescence marker *p16*^*INK4a*^ following loss of the PRC1-mediated repressive H2A-lysine 119 ubiquitination mark. Upon injury, they fail to activate (absence of *Myod* expression) and transit to a full senescent state that is marked by increased *p16*^*INK4a*^ expression, increased number of γ-H2AX foci indicating DNA double strand breaks, and appearance of a senescence-associated β-galactosidase activity. Interestingly, this pre-senescent phenotype could be reverted by silencing of *p16*^*INK4a*^ but not by exposure to a youthful environment, underscoring the cell-autonomous nature of this pre-senescent phenotype.^49^ More recently, it was shown that aged satellite cells display decreased autophagy and mitophagy activities, leading to increased intracellular reactive-oxygen species.^41^ Notably, scavenging ROS restored *p16*^*INK4a*^ repression by re-establishing its repressive H2A-lysine 119 modification, decreased senescence and increased proliferation potential, while restoring autophagy reversed the senescence phenotype and restored the regenerative capacity of geriatric satellite cells.^41,92^ More generally, it appears that cellular quiescence protects satellite cells from ROS through upregulation of genes involved in the antioxidant response, such as *Thioredoxin reductase 1*, *Sulfiredoxin* and *Glutathione peroxidase 3*.^66^

The FGF2/SPRY1 axis appears to be deregulated in aged muscles, yet it remains unclear how this signalling effects its action. In one study, maintenance of aged myofibres in basal medium resulted in compromised proliferation of satellite cells unless FGF2 was added exogenously.^88^ Satellite cells express FGF receptors 1 and 4, and FGFR1 appears as their functional FGF receptor, as *Fgfr4* did not rescue satellite cell proliferation upon *Fgfr1* deletion.^99^ Additional FGF ligands might be involved in the regulation of satellite cell proliferation in vivo, such as FGF6.^100^ In contrast, a decline in satellite cell numbers in the aged niche was attributed to increased FGF2 production and decreased *Spry1* expression.^39^ *Spry1* is a tyrosine kinase receptor that is expressed in quiescent satellite cells, and it antagonises FGF2 signalling. Satellite cell deletion of *Spry1* during regeneration prevents their return to quiescence, resulting in a decrease in the quiescent satellite cell pool after regeneration.^48^ Moreover, aged human satellite cells exhibit higher levels of DNA methylation (mC), and in this context, the *SPRY1* promoter region was reported to have higher levels of mC compared to myogenic cells isolated from young individuals, thereby linking DNA methylation to increased sensitivity to higher levels of FGF2 in the aged niche.^39,101^ Impaired response to FGF2 signalling that results in elevated levels of p38α and p38β diminishes self-renewal capacity of aged satellite cells, and pharmacological inhibition of this pathway,^54,55^ in combination with culture on soft hydrogel substrates^55^ leads to rescue of this phenotype.

Similarly, it has been observed that satellite cells attach to their surrounding microenvironment preferentially via fibronectin/integrin interactions. Aged muscles display substantially lower levels of fibronectin, leading to a weaker adhesion of satellite cells and an increased susceptibility to anoikis,^45^ a phenotype similar to loss of β1-integrin function in SCs.^47^

Some of the decline in satellite cell function has also been attributed to deregulation of the Janus kinases (JAKs, via cytokine receptors), and Signal Transducer and Activator of Transcription proteins (STATs, relay of JAKs to activate nuclear targets) pathway, where STAT3 is stimulated by inflammatory cytokine associated with disease and ageing in muscle. Knockdown of *Jak2* or *Stat3* results in increased satellite stem cell divisions, and transient pharmacological inhibition of this pathway ameliorates transplantation potential and muscle force following injury.^102,103^

An apparent contradiction arises from the above-mentioned studies, regarding whether aged satellite cells can be rejuvenated in a non-cell-autonomous manner. Indeed, geriatric satellite cells could not reverse their pre-senescent state when transplanted into a young recipient muscle,^49^ whereas decreasing niche-derived FGF activity^39^ or tissue complementation with exogenous fibronectin^45^ in aged muscles prevented the loss of satellite cells and restored a youthful muscle regeneration respectively. These findings suggest that the aged muscle phenotype can be reversed by exposure to a ‘young’ environment, however, the pre-senescent state of geriatric satellite cells might represent a point of no return. The most compelling evidence for youthful restoration, and the negative impact of circulating molecules in old mice, come from heterochronic parabiosis experiments, where young donor constituents (blood, secreted molecules, etc) transferred to an old host were shown to restore a rejuvenated phenotype in muscle and the nervous system.^104,105^ The identification of effector molecules that potentially mimic this response is an active area of research. The TGBβ member GDF11, which is homologous to Myostatin (GDF8, inhibitor of myogenesis), is one putative candidate, however its precise role remains under debate.^106–108^

## To what extent is the muscle stem cell population heterogeneous?

**Fig. 5:**
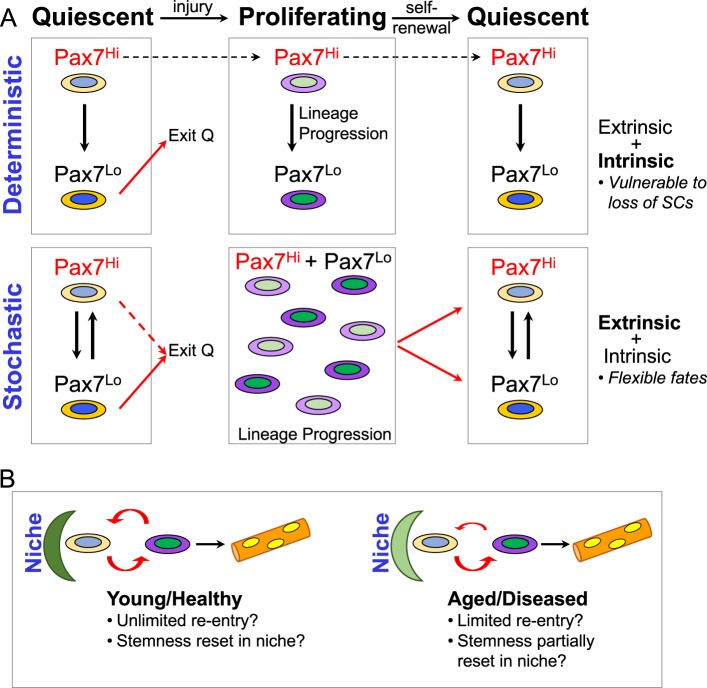
Models for regulation of satellite cells during regeneration. **a** Deterministic model (top), where PAX7^Hi^ cells retain this state through proliferation and return to the quiescent state. Here PAX7^Lo^ cells would derive from PAX7^Hi^ quiescent and proliferating cells and would be poised for commitment. This hierarchical model suggests intrinsic mechanisms as driving forces for maintenance of these relative states and would result in vulnerability if only a subset of the cells in the population have long-term stem-like properties. In the stochastic model (bottom), PAX7^Hi^ and PAX7^Lo^ cells are interchangeable states, presumably due to fluctuations in gene expression, and obedience to extrinsic signals. This model proposes that all satellite cells have the potential to assume a stem-like or committed state. **b** Satellite cells undergo rounds of exit and entry into the niche to assume a quiescent state, and during ageing, exit from the niche is suggested to occur more frequently without replenishment, thereby resulting in declining numbers of muscle stem cells. We entertain the possibility that re-entry into the niche could reset or rejuvenate the stem cell and endow it with properties for long-term persistence

Do proliferating and quiescent cell states each reflect a range of properties that individual myogenic cells can assume, or instead do myogenic cells represent distinct subpopulations each with fixed deterministic potential (Fig. 5)? In this context, it is interesting to note that heterogeneity has been suggested to be a feature of hematopoietic stem cells (HSCs) where at the top of the hierarchy, sub-classes of α and β HSC subpopulations exist with equivalent myeloid potential, whereas α-HSCs have a reduced ability to produce mature lymphoid cells.^109,110^ Cellular plasticity can also underlie heterogeneity where endogenous cells are endowed with greater fate plasticity following their isolation and transplantation, for example, in the blood, skin, and muscle lineages.^111–114^

As indicated above, heterogeneity was also noted among quiescent satellite cells, suggesting that they can explore different levels of quiescence and metabolic states, perhaps driven by local niche signals. Our recent finding that satellite cells from different muscles exhibit different metabolic profiles^115^ suggests that at least some of the heterogeneities reported may be attributed to differences in myofibre niche properties.

Another feature of myogenic cells that is modulated during lineage progression is their response to DNA double strand induced stress. Recent findings have reported that adult stem cells are remarkably proficient in repairing DNA strand breaks compared to their committed progeny.^116–118^ Specifically, satellite cells were reported to repair with greater efficiency and accuracy following irradiation-induced DNA damage, compared to myoblasts and differentiated cells, and that accurate repair depends on the key non-homologous end-joining factor DNA-PKcs (DNA-dependent protein kinase, catalytic subunit).^118^ It remains to be determined if this resistance to DNA damage mediated stress persists in myopathies where satellite cells experience repeated bouts of quiescence and activation, or during ageing, and how this property affects mutational load and genome integrity over time.

Although heterogeneities have been reported among stem cell populations in different tissues, it remains unclear if this reflects a heterogeneity that is associated with distinct cell states, or if the population is uniform, but exhibits a wide range of behaviours. At the organismal level, individuals can survive extreme conditions of temperatures, dehydration and starvation, and some amphibians can undergo multiple consecutive freeze-thaw cycles with no apparent adverse effects.^119^ This can also be the case for individual cells thereby providing the opportunity to adapt to changing environments. However, empirical methods often do not expose these potentials. When cells are analysed, generally post-fixation, their properties several hours before or after the isolation procedure are eclipsed. Single cell transcriptomics can circumvent some of these shortcomings by documenting the range of behaviours in a population, as for example in intestinal and hematopoietic stem and committed cells.^120,121^

Heterogeneity among proliferating cells has been investigated to a limited extent. For example, myogenic cells perform symmetric (SCD) and asymmetric (ACD) cell divisions following muscle injury or after their isolation on single myofibres.^35,40,84,122–129^ It remains unclear what determines this choice, whether it is predetermined, and if SCD and ACD are obligate, or if switching in modes occurs in successive cell cycles. Several types of ACDs have been reported for muscle including non-random DNA segregation (NRDS), where old and new DNA strands segregate to distinct daughter cells,^35,40,84,122,128^ transcription factors that include PAX7 (stem), MYOD (committed) and MYOGENIN (differentiated),^35,84,122,127^ cytoplasmic proteins including NUMB,^122,130^ p38,^129^ and Par proteins and dystrophin.^125^ In addition, several signalling pathways and extracellular molecules have been reported to influence the balance between SCD and ACD including fibronectin and WNT7a.^126,131^

It is clear that cell fates can be governed by extrinsic and intrinsic cues, and studies in *Drosophila* have shown the importance of extrinsic cues in neuroblast and germline cell divisions.^35,132^ Some insights also come from examining individual adult myogenic cells on micropatterns that represent artificial niches, where their shape can be designed to promote SCD and ACD.^35,133,134^ In this scenario, the frequency of NRDS and the asymmetric distribution of PAX7 and MYOGENIN were increased on fibronectin/fibrinogen coated micropatterns with an asymmetric motif compared to those that were placed on a symmetric motif.^35^ Therefore, extrinsic cell adhesion cues can have a major impact on at least two readouts for ACD. If these findings can be extrapolated to the in vivo situation, they suggest that the topology of the immediate microenvironment might dictate cell fate decisions in muscle.

However, these observations pose a conundrum. Although during muscle regeneration the microenvironment is highly dynamic, as far as cell types and extracellular matrix are concerned, a symmetric niche does not clearly exist in vivo; albeit it can be argued that a satellite cell undergoing planar cell divisions on a myofibre during homoeostasis might experience symmetrically distributed extrinsic cues. However, in absence of the myofibre, during G2/M when the axis of cell division will be determined, the resulting daughter cells will most likely experience distinct cues at opposite poles of the division axis in a regenerating tissue. If extrinsic cues have a predominant role in dictating cell fates, then one would expect that all divisions should be asymmetric—but this does not appear to be the case. How can in vitro and in vivo observations be reconciled? One possibility is that cells respond to threshold levels of extrinsic cues, and that an asymmetric response is triggered only if the differential in cell adhesion cues or signalling molecules at opposite poles of the division axis are sufficiently marked. Alternatively, intrinsic differences among cells might lend some to be more responsive to extrinsic asymmetric cues.

Beyond the notion of cellular heterogeneity, it is interesting to ask whether cellular memory persists over consecutive cell divisions. In other words, would a PAX7^Hi^ cell assume a PAX7^Hi^ cell state after one or several cycles of injury and return to homoeostasis, or alternatively, is the internal clock reset with each cell cycle thereby providing stochastic access to both self-renewal and commitment fates to each cell in the population (Fig. 5). Empirical evidence for cell equivalence is lacking in muscle, although this model is currently favoured for stem cells in other tissues.^135–137^

## Muscle regeneration—can it be improved?

Altering genetic or epigenetic functions can lead to compromised regeneration raising the possibility that regeneration in wild-type mice might be improved if these processes can be manipulated. Regenerative myogenesis is non-uniform among mice of distinct genetic backgrounds; SJL/J mice regenerate faster than BALB/c mice following muscle injury or whole muscle engraftments.^138^ Moreover, a milder phenotype is observed with *Mdx* mice on a 129/sv background compared to DBA2 or C57BL/6 backgrounds.^139,140^ Nevertheless, dystrophic mice are generally less severely affected compared to the DMD condition in human.^141^

Strikingly, the MRL multi-strain mouse has been reported to be a “super-regenerator” compared to C57BL/6 for tissue recovery following an ear punch assay.^142,143^ When crossed with γ-Sarcoglycan null mouse, fibrosis and regeneration were reduced in skeletal and cardiac muscle, a phenotype that was mapped in part to a region on Chromosome 2.^144^ Metabolism was suggested to be a driver of this phenotype as the MRL mouse relies more on aerobic glycolytic energy metabolism, increased glutamate oxidation, and reduced fatty acid oxidation compared to C57BL/6 mice.^142^

A notable example of how metabolism can impact disease is provided by the role of Nicotinamide adenine dinucleotide (NAD^+^). Sirtuins consume NAD^+^ and generate nicotinamide for deacetylation of proteins. Additionally, PARP (poly[adenosine 5′-diphosphate (ADP)-ribose] polymerase) proteins consume NAD^+^ during poly(ADP)-ribosylation of proteins. Interestingly, repletion of NAD^+^ was shown to provide protection from metabolic diseases, mitochondrial dysfunction and necrosis, and resulted in improved skeletal and cardiac function in *Dystrophin* and *Dystrophin:Utrophin* mutant mice.^145^ This is thought to occur by countering increased PARP consumption of NAD^+^, thereby leading to the recovery of NAD^+^-dependent sirtuin signalling. In another study, treatment of mice with the NAD^+^ precursor nicotinamide riboside was shown to induce the mitochondrial unfolded protein response, and this resulted in reduced senescence and increased lifespan in mice.^146^ Furthermore, satellite cells undergo a metabolic reprogramming upon in vitro activation after isolation, transiting from fatty acid oxidation to glycolysis as a main source of energy production.^147^ This is accompanied by decreased levels of NAD^+^, which directly regulates the activity of the histone deacetylase SIRT1. This in turn results in increased levels of histone acetylation and a subsequent activation of muscle gene transcription. Satellite cell-specific inactivation of *Sirt1* led to their precocious activation and differentiation.^147^

In another study, examination of foetal, perinatal, quiescent (young, post-mortem, aged) and regenerating myogenic stem cells identified striking differences in metabolic requirements.^115^ Specifically, aged satellite cells were shown to exhibit a compromised oxidative phosphorylation response, relying more on glycolysis for ATP production. In addition, proliferating foetal and perinatal myogenic cells have a low respiration demand, relying more on glycolysis compared to proliferating cells isolated from regenerating muscle, thereby underscoring the impact of the microenvironment on satellite cell metabolic response.

The AMP-activated protein kinase (AMPK) has several roles in metabolism including catabolism and activation of mitochondrial biogenesis. Interestingly, the key metabolic regulator *AMPKα1* was shown to have a major role in controlling the balance between self-renewal and differentiation during muscle regeneration.^148^ Satellite cell-specific inactivation of *AMPKα1* not only increased their glycolytic activity but also drastically enhanced their self-renewal, leading to impaired muscle regeneration. This study places metabolism as a key parameter controlling satellite cell fate decisions.

As the metabolic activity of stem and progenitors is explored in more detail, it is important to note that this data is generally obtained from cells examined ex vivo, where culture conditions potentially alter metabolic profiles. Although relative comparisons have been highly informative, a key future objective is to generate in vivo readouts to assess directly the metabolic demands of stem and niche cells.

Reactive-oxygen species (ROS) have also been linked to a certain extent to pathological processes in regeneration. As indicated above the regeneration deficit when *Numb:Numbl* are conditionally inactivated in satellite cells can be rescued by administering the antioxidant *N*-acetyl cysteine (NAC). However, exposure to NAC does not improve the regenerative process in wild-type mice suggesting that oxidative stress is neither significantly promoting, nor deleterious to the regeneration process at early stages.^93^ During muscle differentiation, there is a higher demand for oxidative metabolism requiring mitochondrial biogenesis and increased ROS production.^92^ In another example, adult long-term-haematopoietic stem cells (LT-HSCs) use hypoxia induced glycolysis preferentially,^149^ perhaps to reduce ROS since these cells, as well as neural stem cells that do so through the action of *FoxO*,^150^ are sensitive to ROS levels that promote differentiation and apoptosis. It has been suggested that elevated levels of ROS drives stem cells out of quiescence in hypoxic conditions, and into proliferation when in normoxia, and that oxidative phosphoryation is low in LT-HSCs.^151^

Conditional inactivation of the transcription factors *Pitx2:Pitx3* that act downstream of *Pax3* and *Pax7* results in elevated levels of ROS and compromised prenatal myogenesis, a phenotype that can be rescued by administration of NAC.^152^ The generation of ROS alone does not account for all of the oxidative stress. Indeed reactive nitrogen species (RNS; reaction of nitric oxide (NO) with superoxide (O_2_^−^) to produce peroxynitrite (ONOO^−^)) is often ignored when considering overall oxidative stress (combined ROS + RNS). The combined actions of ROS and RNS results in modifications of protein structure, lipids, signalling and cytoskeletal elements and DNA damage.^92^

Autophagy is also intimately linked with metabolic processes. Here, damaged proteins and organelles are degraded by lysosomes to assure quality control. In conditions of limiting nutrients, as well as in quiescent cells, breakdown products resulting from autophagy can provide fuel for cellular activities.^41,92,153^ It is clear that autophagy, as well as mitophagy, impact on cell and tissue response during stress in undamaged and injured muscles.^41^ In satellite cells, impairment of autophagy results in loss of proteostasis and increased oxidative stress due to mitochondrial dysfunction, resulting in senescence entry.^41^ These processes need to be examined in stromal cells to ascertain their global impact on muscle physiology.

In the context of myopathies where chronic cycles of degeneration and regeneration prevail, one key aim has been to improve the efficiency of the regeneration process. However, recent findings on *Nfix* mutant alone or on the myopathic *α-Sarcoglycan* null model showed that delaying the regeneration process and shifting to slow myogenesis has a dramatic positive outcome in functional tests including extensive running.^154,155^ Although *Nfix* deletion could act through other pathways unrelated to regeneration speed *per se*, these findings suggest that multiple pathways, including metabolic processes, could be targeted to temper rather than accelerate muscle regeneration.

## The immune response in regeneration and disease

The immune response during regeneration has become another central focus in recent years where monocytes, macrophages, neutrophils and T cells impact on the regeneration process. The critical role of macrophages in wound repair and muscle regeneration has been documented extensively.^156^ A pro-inflammatory response following production of macrophages from infiltrating monocytes, as well as resident macrophages, is associated with phagocytosis and clearing of debris in injured muscle. A second phase of inflammation that follows after a few days is associated with increased myofibre differentiation, angiogenesis, matrix remodelling and subsequently homoeostasis. The distinction between yolk sac and foetal liver derived macrophages raises the question of their respective roles and their dynamics during homoeostasis and regeneration.^157,158^ Furthermore, chronic myopathies are characterised by a non-synchronous regeneration process resulting in the biphasic macrophage response being disrupted, and the coexistence of these populations contributing to the pathophysiology.^156^

Eosinophils and regulatory T cells also infiltrate the damaged muscle where they affect FAP and myogenic cell proliferation, respectively.^159–161^ Notably, regulatory T cells (Treg) produce Amphiregulin that was reported to improve the regeneration process by acting on myogenic cells.^159^ The inflammatory cytokine Prostaglandin E2 (PGE2) was also shown to stimulate satellite cell proliferation and enhance muscle regeneration by promoting the cAMP/phosphoCREB pathway and activation of the NURR1 transcription factor.^162^ Intriguingly, in addition to their pro-myogenic effects, restorative macrophages were shown to stimulate myogenesis/angiogenesis through secretion of Oncostatin M,^163^ whereas this compound is secreted by muscle fibres and functions to maintain satellite cell quiescence.^81^

As for myogenic cells, AMPKα1 has a key role in macrophages where it is required for the anti-inflammatory/restorative inflammatory phenotype during skeletal muscle regeneration. Loss of function in myeloid cells results in lasting inflammation and defects in muscle regeneration due to a block in the phenotypic transition of macrophages.^161^

In summary, recent studies on regenerative myogenesis have underscored the flexibility and wide range of responses exhibited by muscle satellite cells to a variety of stresses, by regulating their depth of quiescence, mode of proliferation and modulation of metabolic processes. Whether return to the quiescent state allows stem cells to reset their state, or perhaps even restore their potential, needs to be explored (Fig. 5b). As the study of quiescence takes more prominence, we note that the mirtron miR-708 was recently shown to regulate quiescence and self-renewal by antagonizing cell migration through targeting the transcripts of the focal-adhesion associated protein Tensin3^164^. Thus, the G0 state exhibits multiple safeguards that merit future attention. Furthermore, the nature of the niche stromal cells is distinct spatially (ex. head vs. trunk) and temporally, pointing to a dynamic niche during prenatal life, adult, disease and ageing. These factors will likely have a significant impact on muscle stem cell properties, as well as their regenerative potential. Future research in these areas should provide valuable information on how to optimise regeneration and boost stem cell potential.
